# Cannabinoid receptor agonist attenuates angiotensin II–induced enlargement and mitochondrial dysfunction in rat atrial cardiomyocytes

**DOI:** 10.3389/fphar.2023.1142583

**Published:** 2023-04-11

**Authors:** Danielle I. Altieri, Yoram Etzion, Hope D. Anderson

**Affiliations:** ^1^ College of Pharmacy, Rady Faculty of Health Sciences, University of Manitoba, Winnipeg, MB, Canada; ^2^ Canadian Centre for Agri-Food Research in Health and Medicine (CCARM), Albrechtsen Research Centre, St Boniface Hospital, Winnipeg, MB, Canada; ^3^ Cardiac Arrhythmia Research Laboratory, Department of Physiology and Cell Biology, Ben-Gurion University of the Negev, Beer-Sheva, Israel; ^4^ Regenerative Medicine and Stem Cell Research Center, Ben-Gurion University of the Negev, Beer-Sheva, Israel

**Keywords:** atrial fibrilation (AF), cardiomyocytes (CMs), angiotensin II, cannabinoid (CB) receptor 1, cannabinoid receptor (CB(2)), cannabinoid

## Abstract

Pathological remodeling of atrial tissue renders the atria more prone to arrhythmia upon arrival of electrical triggers. Activation of the renin-angiotensin system is an important factor that contributes to atrial remodeling, which may result in atrial hypertrophy and prolongation of P-wave duration. In addition, atrial cardiomyocytes are electrically coupled *via* gap junctions, and electrical remodeling of connexins may result in dysfunction of coordinated wave propagation within the atria. Currently, there is a lack of effective therapeutic strategies that target atrial remodeling. We previously proposed that cannabinoid receptors (CBR) may have cardioprotective qualities. CB13 is a dual cannabinoid receptor agonist that activates AMPK signaling in ventricular cardiomyocytes. We reported that CB13 attenuates tachypacing-induced shortening of atrial refractoriness and inhibition of AMPK signaling in the rat atria. Here, we evaluated the effects of CB13 on neonatal atrial rat cardiomyocytes (NRAM) stimulated by angiotensin II (AngII) in terms of atrial myocyte enlargement and mitochondrial function. CB13 inhibited AngII-induced enhancement of atrial myocyte surface area in an AMPK-dependent manner. CB13 also inhibited mitochondrial membrane potential deterioration in the same context. However, AngII and CB13 did not affect mitochondrial permeability transition pore opening. We further demonstrate that CB13 increased Cx43 compared to AngII-treated neonatal rat atrial myocytes. Overall, our results support the notion that CBR activation promotes atrial AMPK activation, and prevents myocyte enlargement (an indicator that suggests pathological hypertrophy), mitochondrial depolarization and Cx43 destabilization. Therefore, peripheral CBR activation should be further tested as a novel treatment strategy in the context of atrial remodeling.

## 1 Introduction

Atrial remodeling can occur due to multiple factors including age, genetic predisposition, and underlying cardiovascular disease (Heijman et al., 2014). Cardiovascular comorbidities including hypertension, heart failure, and valvular disease contribute to the development of atrial remodeling (Chiang et al., 2012). These underlying cardiovascular risk factors result in atrial remodeling making tissue vulnerable to triggers, re-entry, and ectopic firing, thus initiating arrhythmic episodes (Heijman et al., 2014; Forrester et al., 2018). Current pharmacotherapies for some of the consequences of atrial remodeling (such as atrial fibrillation) focus on maintenance of heart rate and rhythm through ion channel blockade. However, they are only modestly effective and can paradoxically lead to the development of life-threatening arrhythmias as well as additional side effects. (Dobrev and Nattel, 2010). Patients with underlying cardiovascular diseases are prone to disease progression beyond atrial remodeling. Enhanced activation of the renin-angiotensin system (RAS) in these patients results in structural and electrical atrial remodeling that markedly increases AF substrate (Schotten et al., 2011). Enhanced levels of angiotensin II (AngII) leads to left atrial enlargement and prolongation of P-wave duration, indicative of structural and electrical atrial remodeling (Kim et al., 2017; Forrester et al., 2018; Jansen et al., 2020). Thus, AngII likely plays an important mechanistic role in the development and consequences of atrial remodeling.

In contrast, adenosine monophosphate-activated protein kinase (AMPK) activation during AF appears to improve atrial structural, contractile, and electrical properties by regulating atrial metabolism and adenosine triphosphate (ATP) expenditure (Kim and Young, 2015; Brown et al., 2021). AMPK is a signaling protein sensor that monitors cellular energy level and is present in all mammalian cells, including atrial and ventricular cardiomyocytes (Arad et al., 2007; Harada et al., 2012). The increased activation rate of atrial cardiomyocytes during AF increases metabolism and energy consumption, resulting in depletion of ATP (Mihm et al., 2001; Brandenburg et al., 2016). Compounds that activate AMPK may mitigate the metabolic demands resulting from AF (Harada et al., 2015; Kim and Young, 2015). In this regard, our group has found that cannabinoid receptor (CBR) ligands exert cardioprotective effects in ventricular hypertrophy models *in vitro* and *in vivo* presumably due to activation of AMPK (Lu et al., 2014; Lu et al., 2020) Recently, we also demonstrated that CB13, a dual CBR agonist with limited brain penetration, is an AMPK activator that prevents tachycardia-induced electrical remodeling and improves energy metabolism in the rat atria *ex vivo* (Lee et al., 2021). In addition to activating AMPK, CB13 appears to alter gap the junction, Cx43, a signal conduction protein that connects cardiomyocytes and is important for synchronous atrial contraction (Qiu et al., 2016; Lee et al., 2021). More recently Cx43 has been demonstrated to be a potential downstream signaling mediator of AMPK activation (Qiu et al., 2016). In fact, in the heart, a key upstream kinase involved in regulating AMPK activity is the serine/threonine kinase, LKB1 (Sakamoto et al., 2006). LKB1 activates AMPKα by phosphorylating Thr172 (Hawley et al., 1996; Hong et al., 2003). Inhibiting AMPK by deleting LKB1 leads to atrial enlargement, fibrosis, and fibrillation as well as reduced AMPK and Cx43 (Kim et al., 2015; Ozcan et al., 2015). Thus, CB13, as an activator of AMPK, warrants further study as a potential therapeutic to attenuate pathological remodeling of atrial cardiomyocytes.

In this study, we examined the effect of CB13 on AngII-induced atrial myocyte enlargement and mitochondrial dysfunction, as characteristics of AF, using neonatal rat atrial cardiomyocytes (NRAM). Neonatal rat cardiomyocytes are often used to study cardiac myocyte hypertrophy. They respond to pharmacological (Shubeita et al., 1990; Knowlton et al., 1991) and mechanical stimuli (Sadoshima et al., 1992) with hallmarks (Chien et al., 1991) of hypertrophy that mimic those *in vivo*: changes in cell size, myocyte architecture, protein synthesis, and expression of hypertrophic genes. Our findings demonstrate that CB13 has cardioprotective effects *via* AMPK activation, independently of LKB1, and prevents structural, metabolic and gap junction abnormalities in neonatal rat atrial myocytes.

## 2 Materials and methods

### 2.1 Materials

AngII was from Sigma Aldrich (St. Louis, MO). CB13 (1-Naphthalenyl[4-(pentyloxy)-1-naphthalenyl]methanone), compound C (dosmorphin, (6-[4-(2-Piperidin-1-ylethoxy) phenyl]-3-pyridin-4-ylpyrazolo [1,5-a]pyrimidine)), JC-1 (tetraethylbenzimidazolylcarbocyanine iodide) Mitochondrial Membrane Potential assay kit and Mitochondrial Permeability Transition Pore (mPTP) assay kit were from Cayman Chemical (Ann Arbor, MI). Primary antibodies from Cell Signaling Technology (Danvers, Massachusetts) for phospho-AMPKα (Thr172) (1:1000, cat. 2535), AMPKα (1:1000, cat. 2603), phospho-LKB1 (1:1000, cat. 3482), LKB1 (1:1000, cat. 3047) and Abcam (Toronto, Ontario) connexin 43 (1:7500, cat. ab11370), CB1R (1:500, cat. ab23703), and CB2R (1:5000, cat. ab45942) were used.

### 2.2 Neonatal rat atrial cardiomyocytes

This study was approved by the University of Manitoba Animal Care Committee and follows Canadian Council of Animal Care guidelines. Use of neonatal myocytes is an accepted *in vitro* model for hypertrophy (Bishopric et al., 1987; Bishopric and Kedes, 1991; Yamazaki et al., 1995; Rokosh et al., 1996; Wu et al., 1996; Liang et al., 1997; Adams et al., 1998; Liang and Gardner, 1998; Wang et al., 1998; Liang and Gardner, 1999; Montessuit and Thorburn, 1999; Zhu et al., 2000; Lim et al., 2001; Takemoto et al., 2001; Higuchi et al., 2003; Liang et al., 2003; Anderson et al., 2004). This protocol is adapted from our NRVM protocol (Wu et al., 1989; Lu et al., 2020). Atria were isolated from 1 to 3-day-old neonatal Sprague-Dawley rats and digested at 37°C with 0.1% trypsin and 0.002% DNase in Ca^2+^- and bicarbonate-free Hanks HEPES (CBFHH) buffer with agitation in repeating 5-min cycles. After each digestion cycle the digested cells were suspended in bovine calf serum (BCS) in 50 mL conical tubes on ice; once the 50 mL conical tube was full it was centrifuged at 60 rpm for 15 min to obtain the cell pellet. Cell pellets were filtered through a 100 µm strainer. To distinguish non-cardiomyocytes from NRAM, cells were seeded (i.e., pre-plated) on T75 tissue culture flasks for 75 min in Dulbecco’s Modified Eagle Medium (DMEM) containing 10% cosmic calf serum (CCS) and 1% penicillin-streptomycin (P/S). Pre-plating allowed non-cardiomyocytes to adhere to the tissue culture flask, whereas most NRAM remained suspended in the culture medium. NRAM were collected from flasks and seeded in 0.1% gelatin-coated 6, 12, 24, and 48 well culture plates in 10% CCS DMEM for 24 h. Cells were serum-starved for 24 h prior to experiments/treatments in 0% CCS DMEM.

### 2.3 Treatments

NRAM were rendered quiescent by serum starvation and immediately used for experiments. NRAM were pre-treated for 1 h with CB13 (1 μM) in the presence or absence of chemical inhibitors of AMPK (compound C; 1 μM; 1 h) (Lu et al., 2020). Our selection of concentrations (and time points) is predicated on first, our previous dose response analyses which found that 1 µM of CB agonists attenuated ventricular myocyte hypertrophy, and this was blocked by 0.1 µM of CB antagonists (Lu et al., 2014), and second, reports that sub- or low µM concentrations ablate other effects of endocannabinoids (Underdown et al., 2005; Romano and Lograno, 2006; Lam et al., 2007). In the absence of pharmacological treatment, NRAM were exposed to vehicle controls. Following the 1 h pretreatment, all treatments (compound C and CB13) remained in the culture media for the entirety of the experimental protocol. Atrial cardiomyocyte hypertrophy and mitochondrial dysfunction were stimulated in NRAM by AngII (10 μM; 24 h) (Kim et al., 2017; Jansen et al., 2018; Chen et al., 2020). Incubation times are based on the results of previously reported time course experiments monitoring CB13 effects in ventricular myocytes (Lu et al., 2014).

### 2.4 Cell surface area measurements

NRAM were cultured in 12-well plates (1.5×10^6^ cells/mL) and treated as required. NRAM were fixed with 4% paraformaldehyde, followed by permeabilization with phosphate-buffered saline (PBS) containing 0.1% Triton X-100 and blocked with PBS containing 2% bovine serum albumin (BSA), as previously described (Anderson et al., 2004). NRAM were incubated with mouse anti-rat sarcomeric α-actinin primary antibody overnight. NRAM were washed three times with PBS and incubated with Alexa Fluor^®^ 488-conjugated secondary anti-mouse IgG1 antibody (ThermoFisher Scientific) for 1 h at room temperature. After washing with PBS, NRAM were viewed using an Olympus IX81 brightfield fluorescence microscope. Surface areas of individual cells were quantified using ImageJ software.

### 2.5 Mitochondrial membrane potential (ΔΨ_m_) imaging

To assess relative changes in mitochondrial membrane potential (ΔΨ_m_) in NRAM, the JC-1 Mitochondrial Membrane Potential assay kit was used, as per the manufacturer’s protocol (Cayman Chemical), and as previously described (Lu et al., 2020). JC-1 is a lipophilic fluorescent reagent; in healthy mitochondria with high ΔΨ_m_, JC-1 concentrates as J-aggregates and emits red fluorescence. In mitochondria with reduced ΔΨ_m_, JC-1 is monomeric and emits green fluorescence.

NRAM were cultured in 24-well plates (0.75 × 10^6^ cells/well). Following treatments, NRAM were incubated with JC-1 for 30 min at 37°C. NRAM images were acquired using an Olympus IX81 brightfield fluorescence microscope. Samples were ex/em at 485/535 nm for monomer green fluorescence and 560/595 nm for aggregate red fluorescence. The ratio of aggregate to monomer (red:green) fluorescence was measured as an indicator of changes in ΔΨ_m_ *via* corrected total cell fluorescence (CTCF) using ImageJ software, where; CTCF = integrated density – (area of cell x mean fluorescence of background) and compared as aggregate CTCF:monomer CTCF.

### 2.6 Mitochondrial permeability transition (mPT) imaging

Mitochondrial Permeability Transition (mPT) Pore assays were performed by measuring mPT pore (mPTP) opening status in NRAM using a commercially available kit (Cayman Chemical, Michigan, United States) according to manufacturer specifications, and as previously described (Lu et al., 2020). This mPTP assay kit utilizes a calcein:cobalt technique that is multiplexed with TMRE. Calcein:cobalt dual staining is a recognized method to assess the degree of mPTP opening. Fluorescence contrast between the mitochondrial puncta and cytosol was measured to quantify mPTP.

NRAM were cultured in 48-well plates (0.5 × 10^6^ cells/well) and treated as required. NRAM were coloaded with calcein, cobalt and TMRE for 15 min, followed by a 5 min wash with PBS. The wavelengths for calcein ex/em 485/535 and TMRE ex/em 545/57L, approximately. Fluorescence was measured using an Olympus IX81 inverted fluorescence microscope. Fluorescence contrast was determined as the difference in fluorescent calcein intensity between mitochondrial puncta and cytosol using ImageJ. For each replicate, images were captured for 5 cells, and fluorescent calcein intensity was measured in three distinct fields.

### 2.7 Western blotting

NRAM were seeded in 12-well plates (1.5 × 10^6^ cells/well) and treated as required. NRAM lysates were prepared in radioimmune precipitation assay (RIPA) lysis buffer with phosphatase and protease inhibitor cocktails, as previously described (Lee et al., 2021). Protein concentration was determined by bicinchoninic (BCA) assay (Thermo Fisher Scientific, Massachusetts, USA). NRAM samples were loaded on Mini-PROTEAN TGX stain-free gels (Bio-Rad, California, USA) at 10 µg/lane and transferred to PVDF membrane (Bio-Rad). Membranes were blocked and primary antibodies were incubated overnight at 4°C. Primary antibody dilutions are listed under *Materials*. Secondary antibodies were from Cell Signaling. As applicable, membranes were stripped and reprobed. Blots were imaged with Bio-Rad Chemidoc™ MP Imaging system and analyzed using Bio-Rad stain-free technology, Proteins of interest were normalized to total protein on the same stain-free membrane and quantified with Image Lab Software (Bio-Rad, Mississauga, Ontario). Each Western blot represents a single replicate (i.e., an independent experimental day). Within each replicate, NRAM were isolated from 60 rat pups, pooled, and plated. Protein from 3 wells was pooled to achieve sufficient protein amounts for Western blotting.

### 2.8 Statistical analysis

Data are reported as mean ± SEM. Experimental data were analyzed using GraphPad Prism 9 software. Normality assumptions were tested using the Shapiro-Wilk test for normality. For normally distributed data, a one-way analysis of variance (ANOVA) followed by Tukey *post hoc* test for multiple comparisons was performed. A *p*-value of ≤0.05 was considered significant.

## 3 Results

### 3.1 CB13 suppresses AngII-induced myocyte enlargement in an AMPK-dependent manner

**FIGURE 1:**
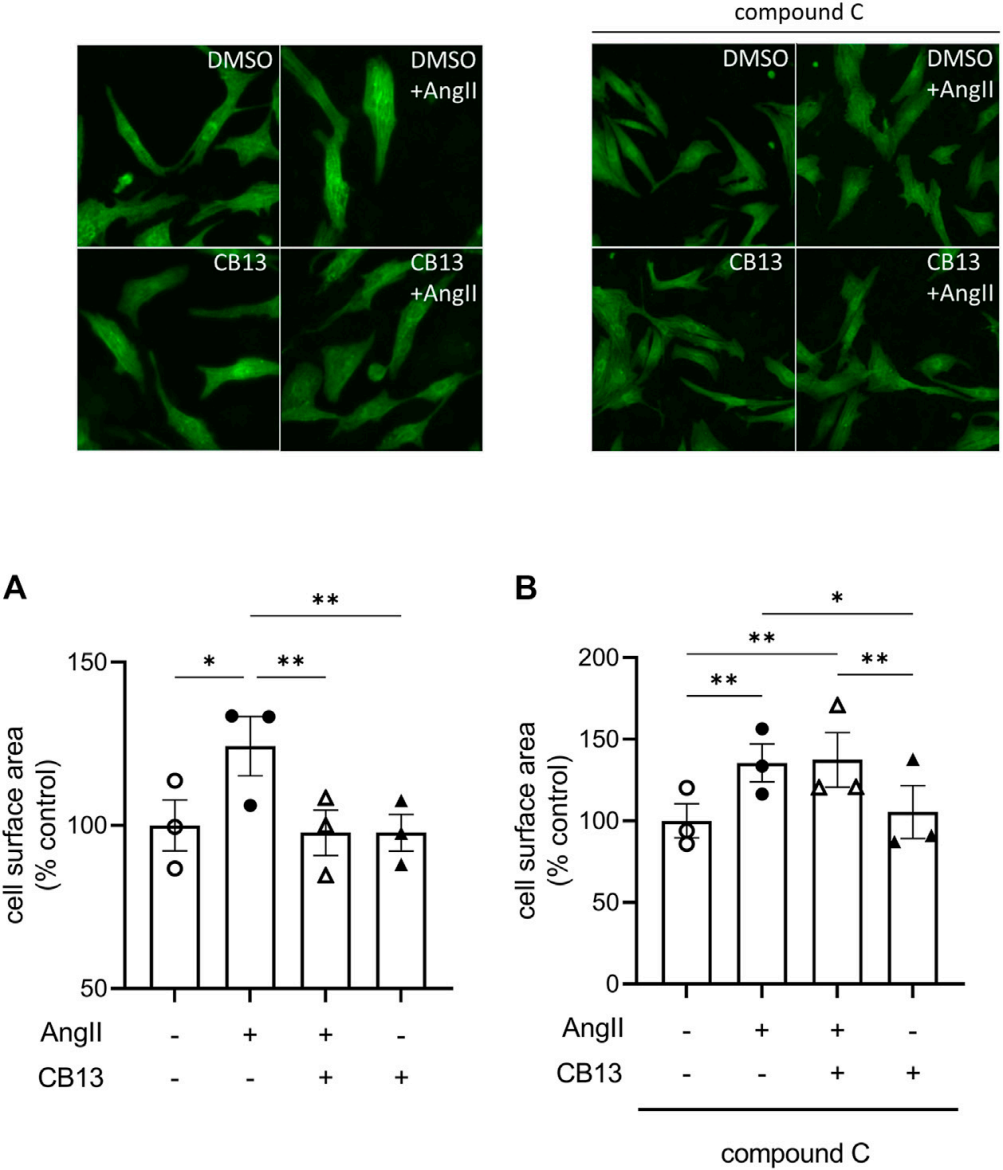
CB13 suppressed AngII-induced enhancement of NRAM cell surface area in an AMPK-dependent manner. **(A)** The ability of AngII to induce NRAM enlargement was abolished by CB13 treatment. **(B)** Treatment of NRAM with compound C, an AMPK inhibitor, prevented the ability of CB13 to suppress NRAM enlargement. Serum-deprived NRAM were pre-treated with CB13 (1 μM, 1 h) in the presence or absence of compound C (1 μM, 1 h), followed by treatment with AngII (10 μM). All treatments remained in media for 24 h. Results are presented with representative fluorescent images. Data are presented as mean ± SEM. n = 3. **p < 0.01.

In patients with cardiovascular diseases, enhanced activation of the RAS system gives rise to atrial remodeling and increased risk for AF (Jansen et al., 2020). Here, we first examined whether CB13 would reduce a hallmark of pathological hypertrophy (that is, increased cell surface area) in atrial cardiomyocytes (Figure 1). Indeed, AngII, increased NRAM surface area compared to control cells (124.6% ± 5.0% vs. control; *p* ≤ 0.01) (Figure 1A). However, CB13 markedly reduced the AngII-dependent NRAM enlargement (97.9% ± 1.5% vs. 124.6% ± 5.0%; *p* ≤ 0.01) (Figure 1A) indicating that CB13 suppresses atrial myocyte enlargement induced by RAS system activation.

We previously demonstrated that CB13 inhibits hypertrophy in ET1-treated NRVM and that disruption of AMPK signaling using a chemical inhibitor, compound C, abolished this effect (Lu et al., 2014). Thus, to determine whether CB13 suppresses enhancement of cell surface area in NRAM through an AMPK-dependent mechanism, we treated cells with compound C (Figure 1B). AngII increased NRAM surface area (136.0% ± 3.5% vs. control, *p* < 0.01). However, the ability of CB13 to prevent AngII-induced enlargement of NRAM was completely abolished by compound C (137.0% ± 4.4% vs. 136.0% ± 3.5%; ns) (Figure 1B). This suggests that CB13 prevents NRAM enlargement in an AMPK-dependent manner.

### 3.2 CB13 prevents AngII-induced mitochondrial membrane depolarization in an AMPK-dependent manner

**FIGURE 2:**
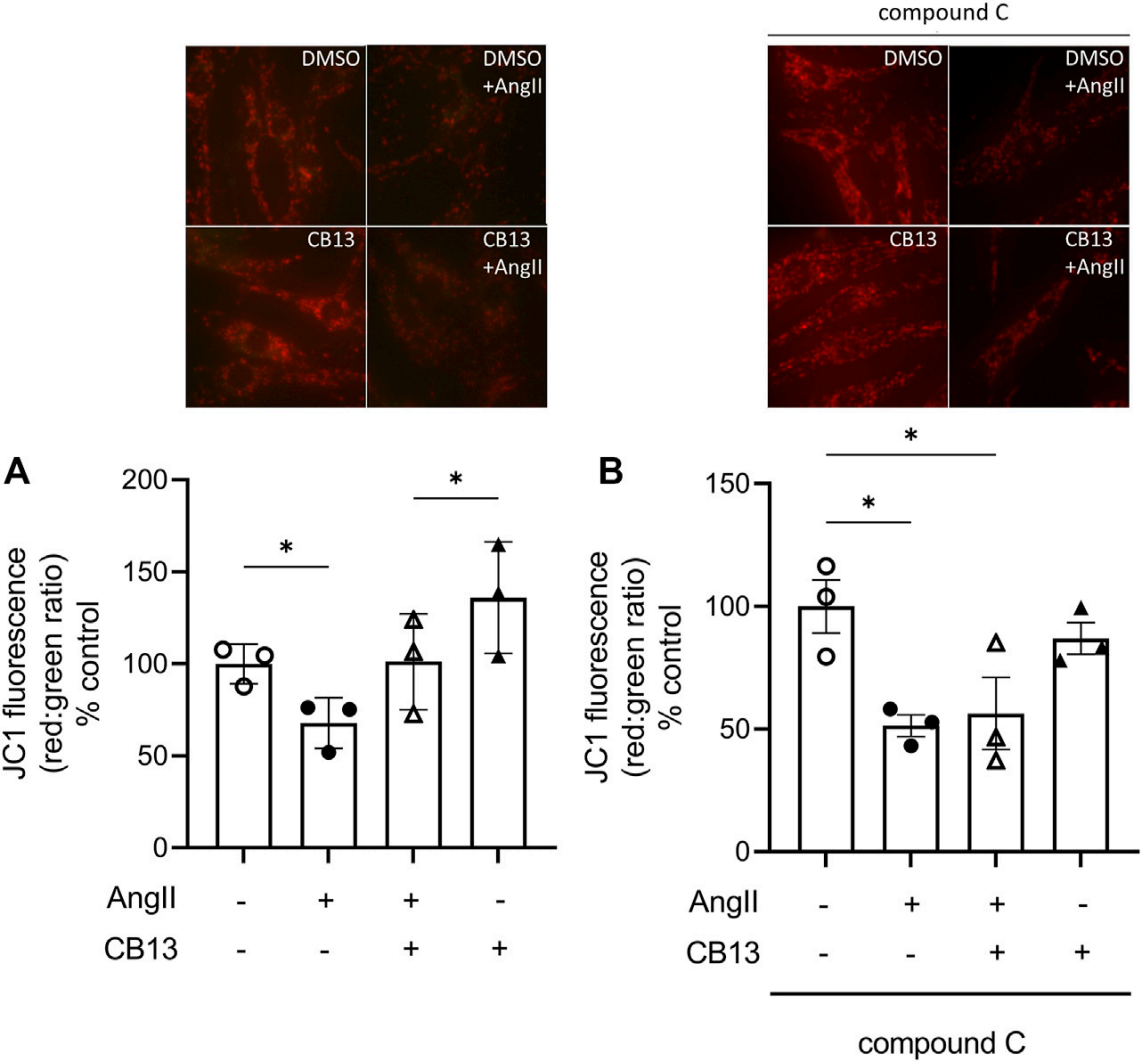
CB13 prevented AngII-induced mitochondrial membrane depolarization in an AMPK-dependent manner. The ratio of red aggregates to green monomers (red:green) reflects membrane depolarization. **(A)** CB13 treatment prevents AngII-induced mitochondrial membrane depolarization. AngII induces membrane depolarization, as indicated by a decrease in red:green, which is attenuated by CB13. **(B)** Compound C, an AMPK inhibitor, prevented the ability of CB13 to alter mitochondrial membrane depolarization. Rescue of red:green by CB13 was prevented by compound C treatment. Serum-deprived NRAM were pre-treated with CB13 (1 μM, 1 h) in the presence or absence of compound C (1 μM, 1 h), followed by treatment with AngII (10 μM). All treatments remained in media for 24 h. Results are presented with representative fluorescent images. Data are presented as mean ± SEM. n = 3. *p ≤ 0.05 and ***p < 0.001.

Mitochondrial dysfunction underlies atrial metabolic remodeling (Harada et al., 2017; Brown et al., 2021). Therefore, mitochondrial membrane depolarization was measured as a parameter of metabolic remodeling. ΔΨ_m_ is determined by the ratio between red J-aggregates to green monomers. The lipophilic and cationic dye, JC-1, selectively enters the mitochondria and changes from green to red as ΔΨ_m_ increases. When cells are healthy, and the ΔΨ_m_ is high, JC-1 forms red J-aggregates. When cells are apoptotic and unhealthy JC-1 remains in monomeric form and will form green monomers. AngII-induced a decline in red aggregate:green monomer (67.3% ± 4.1% vs. control; *p* < 0.05), indicating reduced ΔΨ_m_ (Figure 2A). Mitochondrial membrane depolarization was prevented by CB13 in AngII-treated cells. (100.1% ± 9.4% vs. 67.3% ± 4.1%; *p* < 0.05). When NRAM were pre-treated with compound C, CB13 no longer attenuated the decline in red:green (i.e., mitochondrial membrane depolarization) compared to AngII (59.3% ± 13.8% vs. 58.8% ± 8.7%; ns) (Figure 2B). Therefore, CB13 attenuated AngII-induced mitochondrial membrane depolarization in an AMPK-dependent manner.

### 3.3 Mitochondrial permeability transition pore (mPTP) is not altered by AngII

**FIGURE 3:**
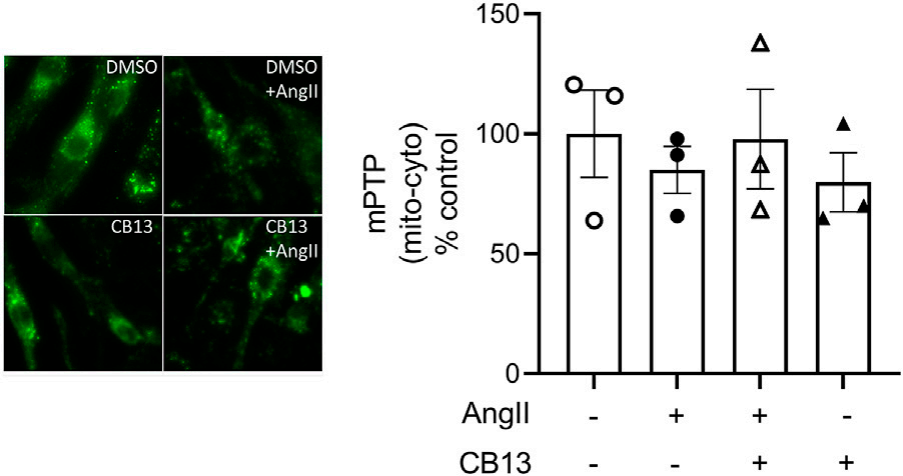
Mitochondrial permeability transition pore (mPTP) was not altered by AngII treatment. NRAM treated with AngII did not undergo changes in mPTP opening. Calcein fluorescence was measured as the contrast between the cytosol and mitochondrial puncta. Serum-deprived NRAM were pre-treated with CB13 (1 μM, 1 h) in the presence or absence of compound C (1 μM, 1 h), followed by treatment with AngII (10 μM). All treatments remained in media for 24 h. Results are presented with representative fluorescent images. Data are presented as mean ± SEM. n = 3.

To measure mPTP atrial cardiomyocytes were coloaded with calcein, cobalt and TMRE and images were acquired. Fluorescence contrast between the mitochondrial puncta and cytosol was measured to quantify mPTP. NRAM treated with AngII did not exhibit alterations in mPTP opening, nor did CB13 influence mPT either in the presence or absence of AngII (Figure 3).

### 3.4 Biochemical effects of CB13 in atrial cardiomyocytes exposed to AngII

**FIGURE 4:**
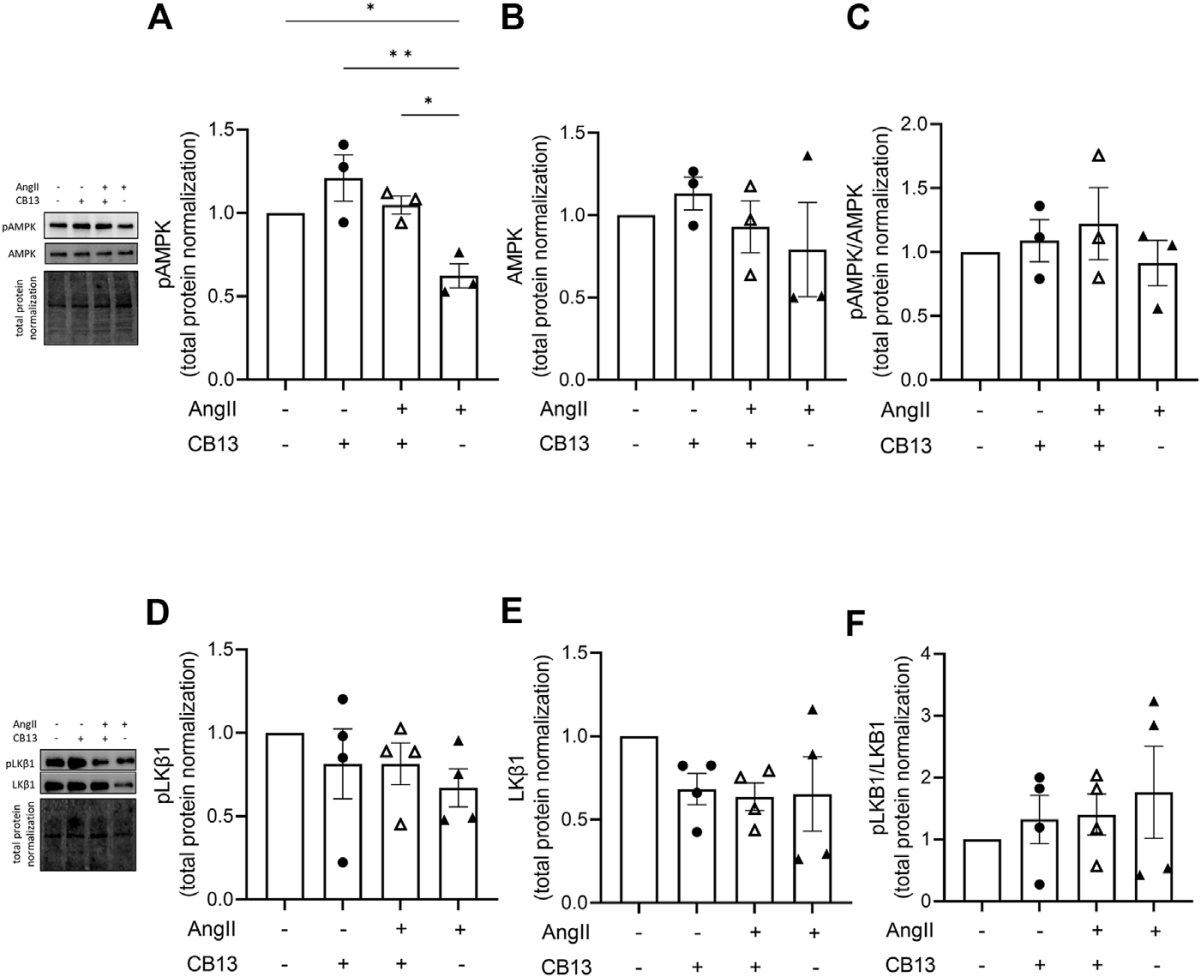
CB13 rescued AngII-induced reduction of phosphorylated AMPK. **(A)** CB13 treatment resulted in phosphorylation of AMPK (normalized by total protein) in NRAM at Thr172 (an indicator of AMPK activation status) compared to control, and rescued AngIIinduced downregulation of p-AMPK. **(B)** Total AMPK and **(C)** pAMPK (when normalized by total AMPK) were unaltered by AngII and CB13 treatment. The proportion of pAMPK in the context of total AMPK doesn’t change (panel C), in part because there is seemingly a trend, albeit statistically insignificant, toward reduced total AMPK. **(D)** p-LKB1, **(E)** total LKB1, and **(F)** p-LKB1/total LKB1 were unchanged by AngII and CB13. Data are presented as mean ± SEM. n = 3–4. *p ≤ 0.05 and **p < 0.01.

Biochemical analyses of relevant signaling mediators were performed using NRAM protein lysates. We began by investigating phosphorylation of AMPKα at Thr172, an indicator of activation status. AngII treatment decreased phosphorylation of AMPKα (0.62 ± 0.07 vs. control; *p* < 0.05) (Figure 4A left). CB13 treatment abolished the inhibitory effects on AMPKα activation elicited by AngII (1.05 ± 0.05 vs. 0.62 ± 0.07; *p* < 0.05) (Figure 4A left). Neither AngII nor CB13 altered phosphorylation or total expression LKB1 (Figure 4B). Note also there were no changes in protein expression between native AMPKα nor native LKB1 regardless of treatment (Figures 4A, B, right). It should also be mentioned that while the proportion of pAMPK in the context of total AMPK does not change (panel C), this is in part because there is seemingly a trend, albeit statistically insignificant, toward reduced total AMPK. The overall finding remains the same. AMPK signaling is depressed in AngII-treated atrial myocytes.

**FIGURE 5:**
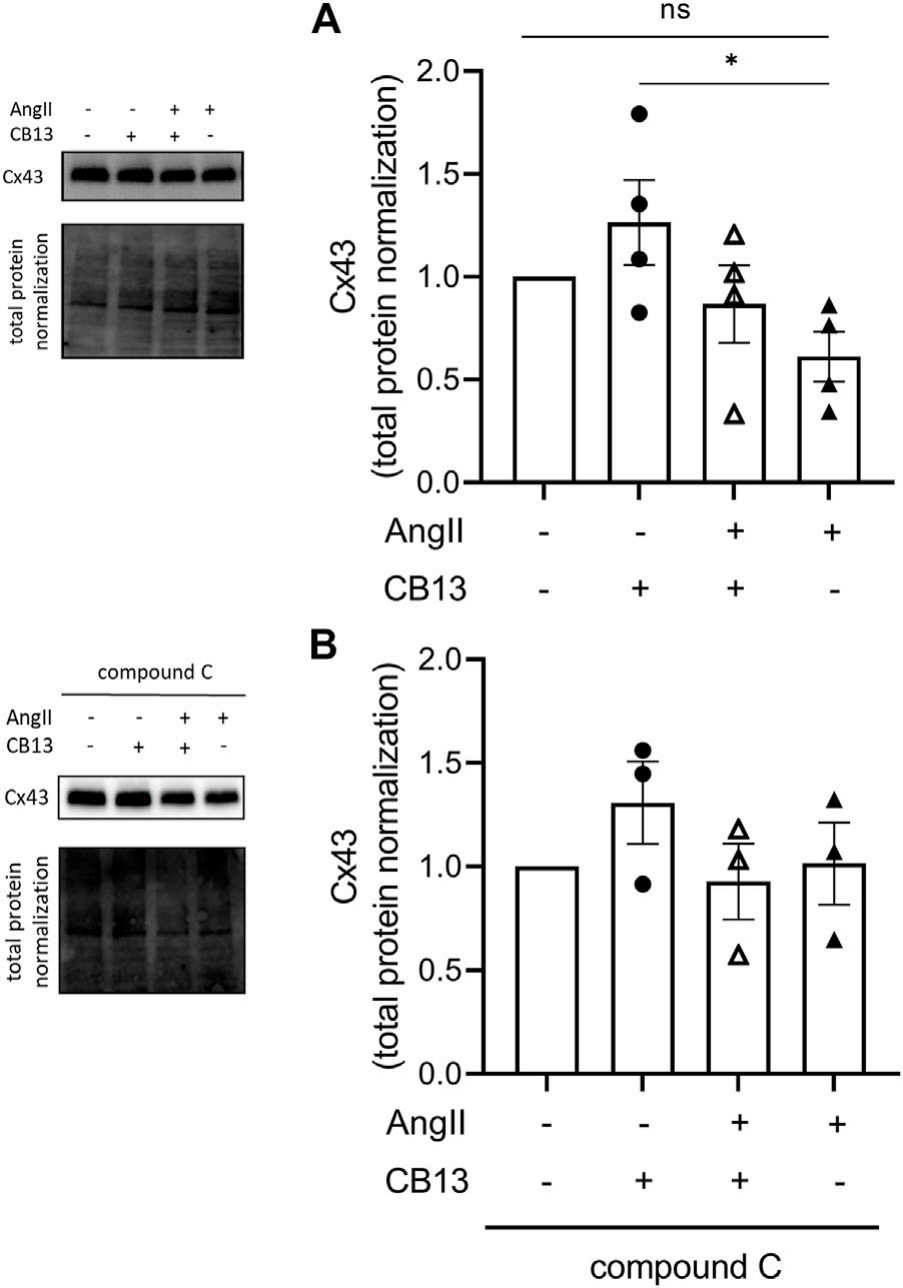
CB13 increased the gap junction, Cx43, compared to AngII controls. **(A)** CB13 increased Cx43 levels compared to NRAM treated with AngII. CB13 also tended to increase Cx43 levels in the presence of AngII, but the latter effect did not reach significance. **(B)** NRAM treated with compound C + CB13 did not have different Cx43 levels when compared to control NRAM. Data are presented as mean ± SEM. n = 3–4. *p ≤ 0.05.

The gap junction, connexin 43 (Cx43), is a major connexin in the heart that mediates cardiomyocyte electrical coupling; underexpression of this key protein is linked to AF (Ozcan et al., 2015). More recently, Cx43 has been associated as a downstream signaling mediator of AMPK (Ahmad Waza et al., 2012; Qiu et al., 2016). AngII tended to inhibit Cx43 expression compared to control and the addition of CB13 tended to restore the AngII-induced reduction of Cx43 levels although both trends did not reach statistical significance. Of note, compared to cells treated with AngII alone, the cells treated with CB13 alone had markedly elevated Cx43 levels and this difference was clearly significant (Figure 5A). NRAM treated with compound C + CB13 did not have different Cx43 levels when compared to control NRAM (Figure 5B).

**FIGURE 6:**
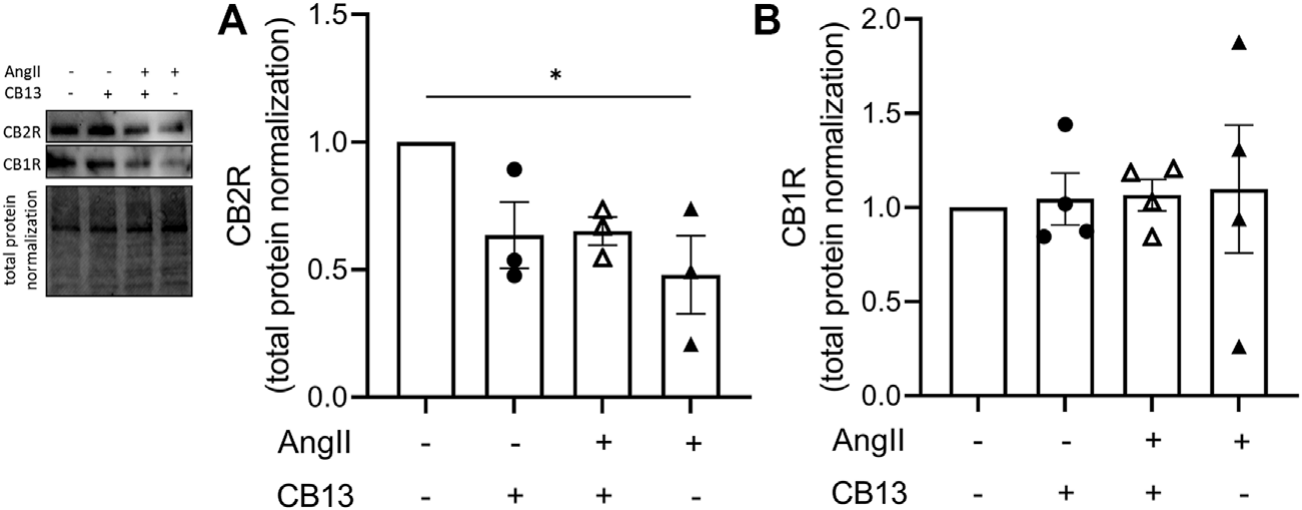
Cannabinoid receptor 2 expression was downregulated in NRAM by AngII. **(A)** CB2R was decreased by AngII-treatment. **(B)** CB1R was unaltered by AngII and CB13. Data are presented as mean ± SEM. n = 3. *p ≤ 0.05.

Lastly, we investigated the expression levels of cannabinoid receptor 2 (CB2R) and cannabinoid receptor 1 (CB1R). CB2R was downregulated by AngII in NRAM (0.48 ± 0.15 vs. control; *p* < 0.05) (Figure 6A). In contrast, CB1R was unaltered by AngII and/or CB13 treatment (Figure 6B).

## 4 Discussion

Our findings demonstrate that the CBR ligand, CB13, attenuates atrial myocyte enlargement and mitochondrial dysfunction in NRAM through AMPK signaling, building upon previous evidence for beneficial effects of CBR ligands in stressed cardiomyocytes (Lu et al., 2014; Lu et al., 2020; Lee et al., 2021). In addition, our data may suggest that AMPK-dependent changes in Cx43 expression also contribute to the possible beneficial effect of CBR ligands. Overall, this study demonstrates three novel findings regarding the effects of CB13 in NRAM: 1) the anti-hypertrophic ability of CB13, previously detected in ventricular cardiomyocytes, likely extends to atrial cardiomyocytes (at least in terms of enhanced cell surface area) and is AMPK-dependent; 2) CB13 inhibits mitochondrial depolarization in a manner dependent on AMPK, but independent of mPTP; 3) AMPK activation (that is independent of LKB1 signaling) and Cx43 are effectors of cardioprotection against AngII-induced atrial stress.

RAS activation appears to play a key role in atrial remodeling and its consequences, particularly in patients with cardiovascular diseases (Allessie et al., 2001; Kim et al., 2017). Increased RAS activation leads to multiple alterations in the atria including hypertrophy and abnormal contractile function of the tissue (Allessie et al., 2001; Schotten et al., 2011). Our findings show that CB13 prevented the AngII-induced increase in cell surface area of NRAM. This finding extends upon our previous results of growth inhibition in NRVM and demonstrate the global nature of beneficial CBR activation in the heart. (Lu et al., 2014). Furthermore, we demonstrated that AMPK inhibition, using compound C blocked the ability of CB13 to attenuate myocyte enlargement. This indicates that the molecular mechanism behind the beneficial effect of CBR activation is logically related to metabolic terms. Furthermore, this finding is consistent with the multiple recent reports regarding the possible beneficial effects of AMPK activation in the context of AF (Ikeda et al., 2009; Harada et al., 2015; Lenski et al., 2015; Brown et al., 2021).

Metabolic stress, leading to a reduction in ATP and an increase in ROS, is an important contributor to atrial remodeling (Brown et al., 2021). This is often exacerbated by remodeling-induced arrhythmia wherein the rapid atrial rate results in energy depletion and acute metabolic stress. This in turn activates AMPK as a compensatory mechanism (Harada et al., 2012; Harada et al., 2015). In fact, the progression from atrial remodeling to persistent arrhythmia has been linked to a decrease in AMPK activation (Brown et al., 2021), and this is improved by interventions that promote AMPK activation h (Harada et al., 2015). Thus, reduced AMPK activation may be an underlying mechanism that permits the deleterious consequences of atrial remodeling. Our findings show activation of AMPK mediates the ability of CB13 to attenuate NRAM enlargement. Our group also recently demonstrated that CBR ligand activation prevented AMPK inhibition and atrial electrical remodeling following atrial tachypacing *ex vivo* (Lee et al., 2021). Overall, the current results, in conjunction with previous reports, support the notion that AMPK activation may be a viable treatment modality in the context of structural and electrical atrial remodeling. Given the apparent safety of CBR activation in hemodynamic terms (Lee et al., 2021), this treatment modality might be an attractive therapeutic option in this regard.

The mechanism by which CB13 activated AMPK is unclear. Although LKB1 activates AMPKα by phosphorylating Thr172 (Hawley et al., 1996; Hong et al., 2003). we found that CB13 failed to activate LKB1 in rat atria *ex vivo* (Lee et al., 2021). One possibility is that AMPK activation is instead achieved by suppression of the AMPK phosphatase, PPM1A. PPM1A reduced AMPK phosphorylation/activation in the presence of aldosterone (Tabony et al., 2011), which is a known hormonal stimulus of AF substrate.

An additional aspect of metabolic remodeling is mitochondrial dysfunction. ΔΨ_m_ was reduced in AngII-treated NRAM, indicating a decline in proton motive force and an overall reduction in free energy available to generate ATP (Brown and O'Rourke, 2010). However, ΔΨ_m_ was restored to control levels by CB13 in an AMPK-dependent manner. This contrasts with the AMPK-independent ability of CB13 to prevent reduced mitochondrial depolarization in NRVM (Lu et al., 2020). However, this finding was likely not due to complete lack of AMPK involvement but rather simultaneous loss of CBR/AMPK effect on the mitochondrial electron transport chain (Lu et al., 2020).

The role of mPT in cardiovascular injury is well-studied, as opening of mPTPs results in apoptosis and cell death in myocardial infarction and left ventricular dysfunction (Minners et al., 2000; Clarke et al., 2002; Hausenloy et al., 2002; Argaud et al., 2004; Hausenloy et al., 2004; Argaud et al., 2005; Oka et al., 2008). However, in the context of arrhythmia, the role of mPTP activity remains unclear (Brown and O'Rourke, 2010). While the collapse in ΔΨ_m_ has been demonstrated in numerous studies and leads to arrhythmias (Romashko et al., 1998; Huser and Blatter, 1999; Zorov et al., 2000; Aon et al., 2003; Lu et al., 2020), the mechanistic basis is poorly understood (Brown and O'Rourke, 2010; Akar et al., 2005; Brown et al., 2008). AngII-induced atrial mitochondrial membrane depolarization is not linked to mPT, as opposed to the ΔΨ_m_-mPTP coupling in ET-1-treated NRVM. The contradictory involvement of mPTP in CB13-treated NRAM but not CB13-treated NRVM is possibly explained by functional differences of ion complexes within the inner membrane that are involved in ΔΨ_m_. For example, blocking the inner membrane ion channel, IMAC, was protective against arrhythmias by preventing ΔΨ_m_ reduction, improving left ventricular developed pressure, and preventing action potential shortening (Billman et al., 1998; Aon et al., 2003; Brown et al., 2008). As noted above, we recently reported that CB13 treatment prevents atrial refractoriness shortening in an *ex vivo* rat model exposed to atrial tachypacing, suggesting prevention of action potential shortening by CB13 (Lee et al., 2021). It is plausible that CB13 counteracted the tachypacing-related remodeling by modulating ΔΨ_m_ *via* IMAC. However, this possibility should be further confirmed in future studies. Overall, AngII induces mitochondrial dysfunction in NRAM, and utilizing therapeutics that target metabolic alterations and preserve ΔΨ_m_ may prevent the development of arrhythmias.

The aforementioned structural remodeling is often accompanied by metabolic and electrical remodeling (Brown et al., 2021). In addition to AMPK activation, our data suggest that Cx43 may be a downstream effector of CB13. Cx43 is a major gap junction assembled from connexin proteins that are necessary for cell-to-cell electrical communication. Found in both ventricular and atrial myocardial tissue (Severs et al., 2008; Glukhov et al., 2012), Cx43 is downregulated during several atrial remodeling studies (Akar et al., 2007; Yan et al., 2013; Lee et al., 2021). Ozcan *et al.* also demonstrated that aspirin and metformin, both activators of AMPK, preserved Cx43 protein levels and atrial size in an LKB1 knockout mouse model (Ozcan et al., 2020). Our finding that CB13 preserves Cx43 levels offers insight into the potential effect of CB13 in terms of electrophysiological coupling between NRAM. Whether CBR/AMPK crosstalk is required for CB13 effects on Cx43 remain to be elucidated. Cx43 is reportedly a downstream signaling effector of AMPK activation, possibly through K_ATP_ channels (Ahmad Waza et al., 2012; Qiu et al., 2016). Our data show that in NRAM, AngII reduced phosphorylation of AMPK, CB13 rescued AMPK activation, and concomitantly prevented AngII-induced suppression of Cx43. We further demonstrate that CB13 increased Cx43 compared to AngII-treated NRAM, which is consistent with our previous report that CB13 rescued Cx43 in an acute tachypaced atrialmodel. (Lee et al., 2021). This finding supports the notion that Cx43 expression levels are linked to AMPK activation status in a way that may be clinically relevant in the context of atrial remodeling. Moreover, the effect of CB13 on cellular localization of Cx43 is unknown, though there is some evidence that dexmedetomidine, an α2-agonist with antiarrhythmic properties, increased the distribution of Cx43 in the context of ischemic cardiomyopathy *via* AMPK activation. Whether CBR/AMPK signaling likewise affects Cx43 distribution is an interesting question that warrants further study, and therefore this was added to the discussion. Lastly, CB2R was downregulated in atrial cardiomyocytes by AngII. This may suggest that the inhibitory effect of AngII on CB2R is perhaps a route by which AngII prevents AMPK signaling.

In summary, our study demonstrates that CB13 prevents hallmarks of AngII-induced structural, metabolic, and possibly also electrical remodeling in a largely AMPK-dependent manner. Specifically, the CBR/AMPK axis prevents AngII-dependent NRAM enlargement, restores ΔΨ_m_, and may address electrophysiological aberrations by rescuing expression of Cx43. CBR-based interventions therefore warrant further study as a potential therapeutic approach to the clinical problem of deleterious atrial remodeling.

## 5 Limitations

Our study interrogated how CB13 mitigates aspects of atrial remodeling and hypertrophy such as atrial myocyte enlargement using neonatal cardiomyocytes *in vitro*. As neonatal cardiomyocytes are historically viewed as a suitable model for interrogating hypertrophic pathology, as patients with heart failure demonstrate reversion to cardiomyocyte neonatal phenotype during disease progression, our conclusions within this model are justified. However, in the future, this research direction would be strengthened by transitioning to IPSC-derived cardiac myocytes as experimental paradigm. Furthermore, this study is based upon previous findings in *ex vivo* Langendorff intact hanging hearts (Lee et al., 2021). An important next step will be to investigate the effects of CB13 *in vivo* using a novel tachypacing methodology (Lee et al., 2021; Murninkas et al., 2021).

## Data Availability

The raw data supporting the conclusions of this article will be made available by the authors, without undue reservation.
