# Supplementary material for: Cannabinoid receptor agonist attenuates angiotensin II–induced enlargement and mitochondrial dysfunction in rat atrial cardiomyocytes
Source: Front Pharmacol. 2023 Apr 11;14:1142583. doi: 10.3389/fphar.2023.1142583 (PMC10126395; doi:10.3389/fphar.2023.1142583)

Supplement for "*Cannabinoid Receptor Agonist Attenuates Angiotensin II–Induced Enlargement and Mitochondrial Dysfunction in Rat Atrial Cardiomyocytes*"

Supplement 1A – Raw blots for Figure 4A – AMPK and total AMPK

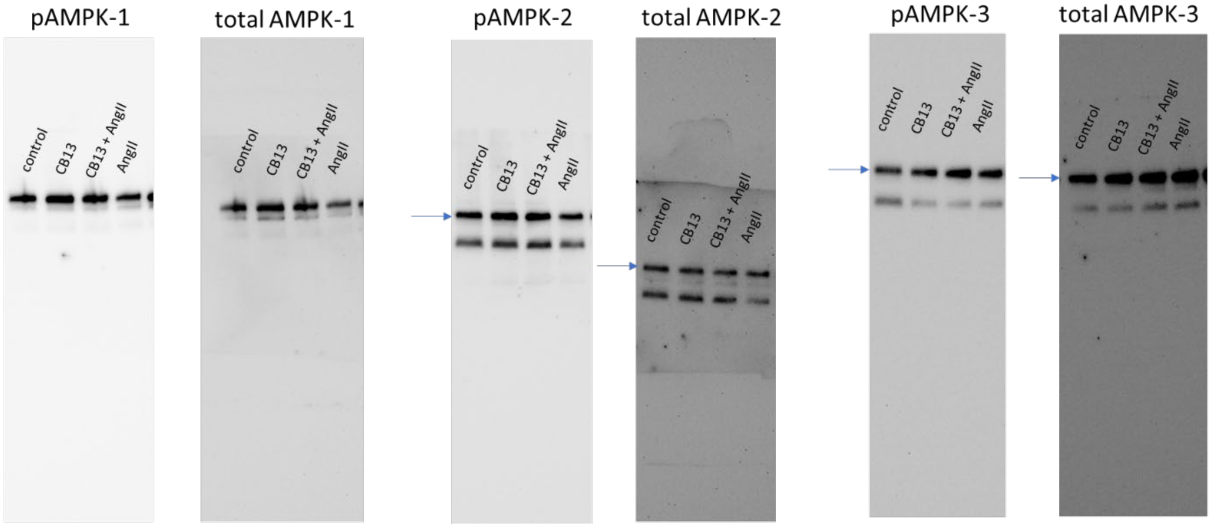

Supplement 1B - raw blots for Figure 5A – Cx43

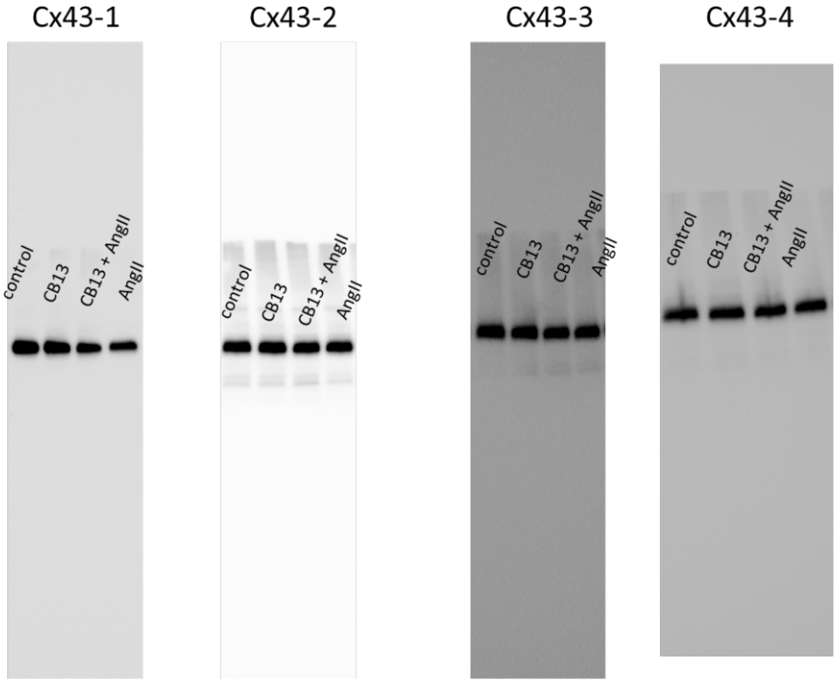

Supplement 1C - raw blots for Figure 5B – Cx43 (Compound C)

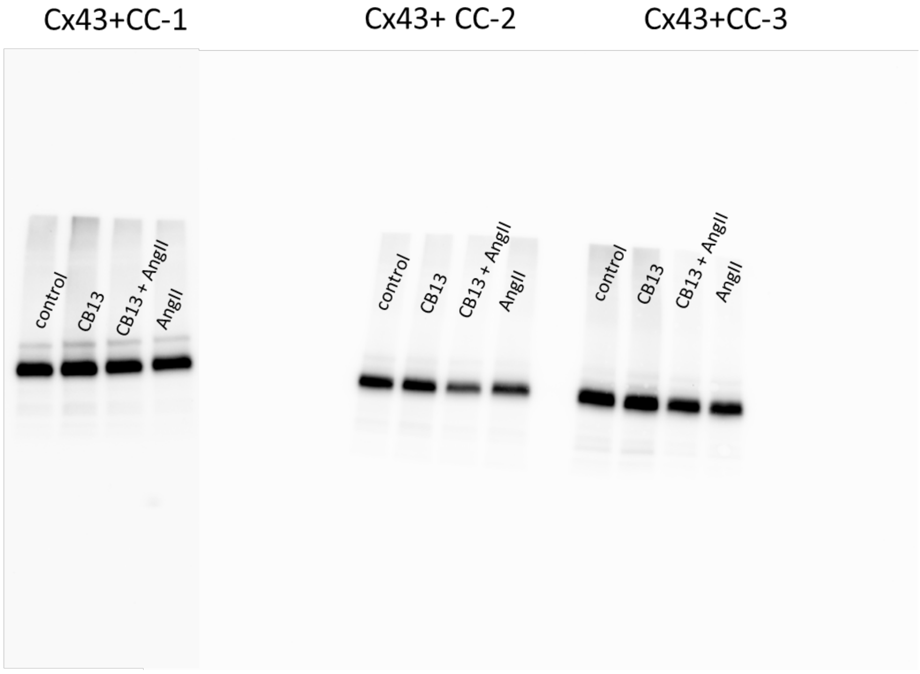

Supplement 1D - raw blots for Figure 6 – CB1R

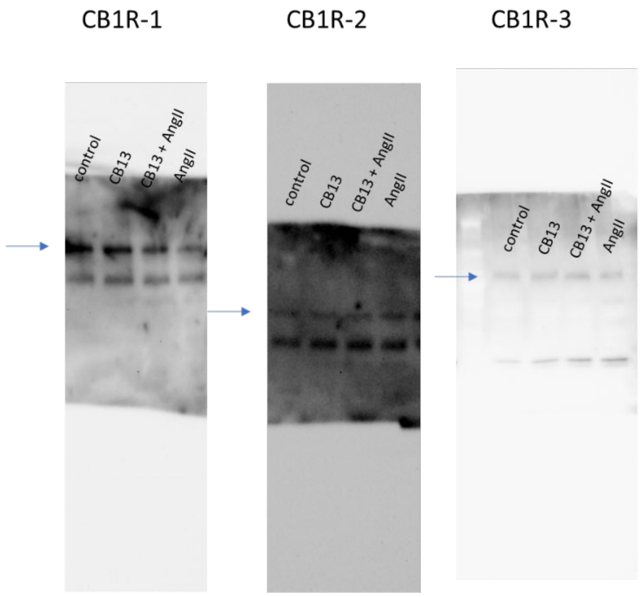

Supplement 1E - raw blots for Figure 6 – CB2R

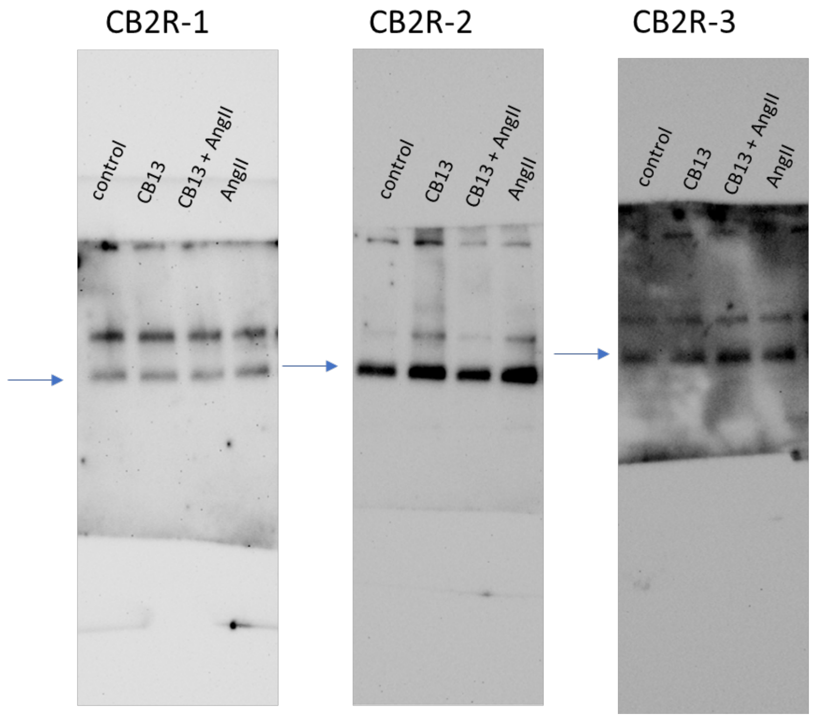

Supplement: Supplementary file 1 [file DataSheet2.PDF]
